# The Transcoelomic Ecosystem and Epithelial Ovarian Cancer Dissemination

**DOI:** 10.3389/fendo.2022.886533

**Published:** 2022-04-28

**Authors:** Sabrina J. Ritch, Carlos M. Telleria

**Affiliations:** ^1^ Experimental Pathology Unit, Department of Pathology, Faculty of Medicine and Health Sciences, McGill University, Montreal, QC, Canada; ^2^ Cancer Research Program, Research Institute, McGill University Health Centre, Montreal, QC, Canada

**Keywords:** ovarian surface epithelium, fallopian tube epithelium, omental metastasis, high-grade serous ovarian cancer (HGSOC), ascites fluid, peritoneal carcinomatosis, transcoelomic dissemination

## Abstract

Epithelial ovarian cancer (EOC) is considered the deadliest gynecological disease and is normally diagnosed at late stages, at which point metastasis has already occurred. Throughout disease progression, EOC will encounter various ecosystems and the communication between cancer cells and these microenvironments will promote the survival and dissemination of EOC. The primary tumor is thought to develop within the ovaries or the fallopian tubes, both of which provide a microenvironment with high risk of causing DNA damage and enhanced proliferation. EOC disseminates by direct extension from the primary tumors, as single cells or multicellular aggregates. Under the influence of cellular and non-cellular factors, EOC spheroids use the natural flow of peritoneal fluid to reach distant organs within the peritoneal cavity. These cells can then implant and seed distant organs or tissues, which develop rapidly into secondary tumor nodules. The peritoneal tissue and the omentum are two common sites of EOC metastasis, providing a microenvironment that supports EOC invasion and survival. Current treatment for EOC involves debulking surgery followed by platinum-taxane combination chemotherapy; however, most patients will relapse with a chemoresistant disease with tumors developed within the peritoneum. Therefore, understanding the role of the unique microenvironments that promote EOC transcoelomic dissemination is important in improving patient outcomes from this disease. In this review article, we address the process of ovarian cancer cellular fate at the site of its origin in the secretory cells of the fallopian tube or in the ovarian surface epithelial cells, their detachment process, how the cells survive in the peritoneal fluid avoiding cell death triggers, and how cancer- associated cells help them in the process. Finally, we report the mechanisms used by the ovarian cancer cells to adhere and migrate through the mesothelial monolayer lining the peritoneum. We also discuss the involvement of the transcoelomic ecosystem on the development of chemoresistance of EOC.

## Introduction

Ovarian cancer is considered the deadliest gynecological disease and the fourth leading cause of cancer death in women worldwide. Epithelial ovarian cancer (EOC) is the most common form of ovarian cancer and is classified into different histotypes. The various subtypes of EOC can be divided into Type 1 and Type 2 disease. Type 1 EOCs typically grow slowly, are diagnosed at early-stage disease (stage I or II) and tend to be TP53 wild type. Type 1 EOCs include mucinous, endometrioid, clear cell, and low-grade serous. Type 2 EOC tend to be quite aggressive, often diagnosed at late-stage disease (stage III or IV) and are typically P53 mutant. High-grade serous ovarian cancer (HGSOC), a type II EOC, is the most aggressive form of the disease and accounts for 70% of all deaths from EOC. HGSOC is most often detected at late stages of the disease when the cancer has already metastasized to distant sites within the peritoneal cavity. Initial treatment with a combination of platinum-based chemotherapy shows promising results in most patients, with 70% of patients responding. However, 95% of patients will eventually relapse with a more severe and chemoresistant disease (1). HGSOC metastasizes from the site of origin by direct extension from the tumor, either as single cells or as multicellular structures (MCS). These MCS use the natural rotational flow of peritoneal fluid to travel through the peritoneal cavity and reach distant sites where they establish themselves and form metastatic lesions (2).

The peritoneal cavity is a closed off compartment in which the abdominal and reproductive organs reside. Organs within this cavity are covered in a protective layer of peritoneal tissue and are suspended in the peritoneal fluid. The peritoneal tissue is composed of an underlying stroma of fibroblasts embedded and dispersed within an extracellular matrix (ECM), primarily composed of collagen I, collagen IV, and fibronectin, and topped with a monolayer of mesothelial cells. The mesothelial cells play a protective role by providing a non-adhesive barrier allowing for the free movement of internal organs. This non-adhesive quality of the mesothelial cell layer is the result of the glycolax. Made up of glycoproteins, the glycolax is produced by the mesothelial cells and functions mainly to maintain hydration and reduce frictional forces against peritoneal organs (3). Under normal conditions, 5-20 mL of peritoneal fluid can be found within the peritoneal cavity. This fluid is mostly composed of ovarian exudate, plasma transudate, retrograde menstruation, and macrophage and mesothelial cell secretions. The peritoneal fluid contains a multitude of immune cells as well as floating mesothelial cells and functions to reduce friction between peritoneal organs, fluid exchange with plasma, and exchange of immune cells. The peritoneal fluid is constantly circulating within the peritoneal cavity in a circular motion, with gravity aiding with downward flow while pressure from respiratory movements lead to upward flow of the fluid. Under pathological conditions, it is common to observe an increase in the peritoneal fluid, often caused by a disruption in the balance between secretion and drainage. This accumulation is called ascites fluid and plays an important role in the dissemination of HGSOC (4).

Throughout the process of dissemination, HGSOC cells encounter varying microenvironments that influence their progression. This review covers how HGSOC cells are influenced by the microenvironments at their site of origin in the secretory cells of the fallopian tube or the ovarian surface epithelium, their survival through the peritoneal fluid, and the adhesion and infiltration into the peritoneum. We also review the mechanisms within this transcoelomic ecosystem that lead to HGSOC chemoresistance.

## Microenvironment at the Site of Origin

**Figure 1:**
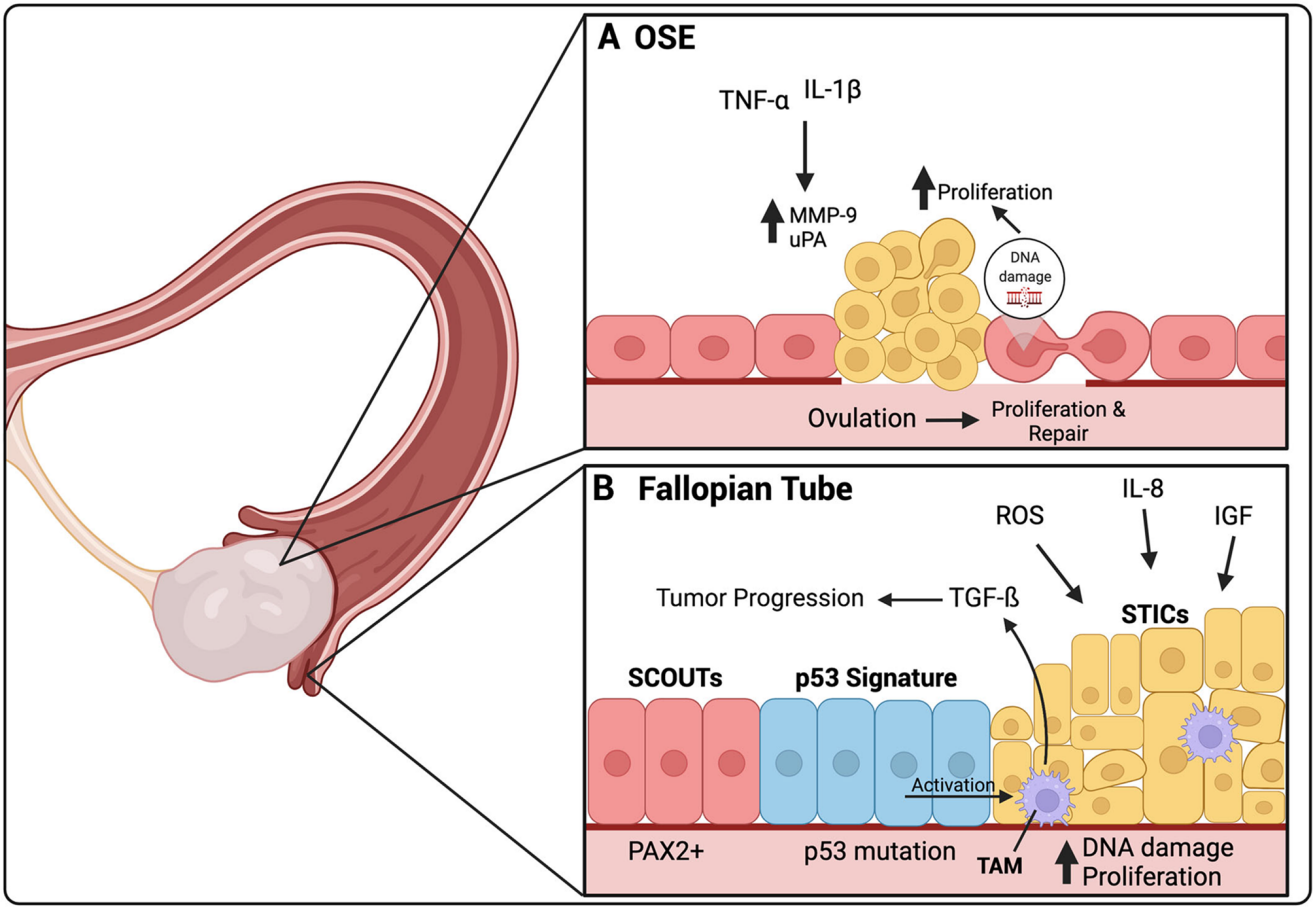
The origin of HGSOC is thought to be either in the secretory cells of the fallopian tube or the ovarian surface epithelium (OSE). **(A)** Ovulation induces a constant damage and repair cycle in the OSE. In certain cases, these constant DNA repair cycles can cause DNA damage, leading to uncontrolled proliferation of the OSE. TNF-α and IL-1β are present in the inflammatory environment created by ovulation. They increase the expression of MMP-9 and uPA, both involved in ECM repair after ovulation, but have also been linked to HGSOC premetastatic lesions. **(B)** SCOUTs, an extended area of secretory cells, are thought to be the initial precursor lesion for HGSOC. SCOUTs then evolve into p53 signature, secretory cells that have developed p53 mutations. STICs are thought to be the immediate precursor lesion of HGSOC. ROS, IL-8 and IGF released during ovulation, are thought to cause increases in DNA damage leading to increased proliferation of STIC and development into HGSOC. Activated TAMs can be found in STIC lesions, secreting TGF-β, which contributes to tumor progression. TAMs are activated by p53 mutant cells within STIC lesions. Created with BioRender.com.

There are five described histotypes of EOC, each thought to have its own site of origin. For instance, mucinous ovarian cancers are considered to originate from the gastrointestinal epithelium, while endometrioid and clear cell are thought to derive from endometriosis lesions. The site of origin of HGSOC has long been debated. Two sites arise as the most likely candidates: the ovarian surface epithelium (OSE) and the secretory cells of the fallopian tubes (**Figure 1**). Both present a unique microenvironment prone to the development of HGSOC, with a multitude of evidence to support each one. The OSE is composed of a single layer of cuboidal epithelial cells covering the surface of the ovary. It is separated by a basement membrane from the underlying ovarian stroma, a collagenous connective tissue. Its main function is to take part in ovulation by rupturing to allow for follicle release, followed by its repair (5). The fallopian tubes are composed of two distinct cell types: secretory cells and ciliated cells. Tubal transport is possible due to the combined function of both of these cell types. Secretory cells produce and secrete a mucosal discharge that aids in the propulsion of an ovum through the fallopian tubes by the ciliary activity of the ciliated cells (6).

### The Ovarian Surface Epithelium

The OSE was long thought to be the site of origin of HGSOC. This was due to the observation of atypical cells found in the OSE, adjacent to ovarian cancer, and the increased presence of cysts found within the ovaries of women with ovarian cancer (7, 8). There is a known reduced risk of EOC associated with long-term oral contraceptive use and pregnancy and number of ovulations in a woman’s lifetime is associated with increased risk (9, 10). Ovulation is linked with a pro-inflammatory environment and continuous repair. This continuous cellular injury and repair is thought to cause impairment of DNA repair mechanisms, particularly in individuals with BRCA1/BRCA2 mutations in which DNA repair mechanisms are already malfunctioning, subsequently leading to increased risk of malignancy (11). Once ovulation is complete, OSE cells, neighboring the site of injury gain the ability to proliferate towards the damaged area to remove old collagen and deposit new collagen proteins. It is thought that the damage to the basement membrane below the OSE, during ovulation, can lead to tumor initiation due to the accumulation of genetic changes that occur after continuous damage and repair. A study of human ovarian tumors demonstrated that premalignant lesions were associated with the loss of the underlying basement membrane, specifically the ECM proteins collagen IV and laminin, and increased proliferation of the OSE cells (12). It was found that exposure to the inflammatory cytokines tumor necrosis factor (TNF)-α and interleukin (IL)-1β increased the expression of matrix metalloproteinase (MMP) 9 and urokinase type plasminogen activator (uPA) by OSE cells (**Figure 1A**). Both are known to be involved in ECM remodeling and basement membrane repair after ovulation. Specifically, MMP9 activation was associated with degradation of collagen IV, an ECM protein linked to premalignant lesions of ovarian cancer (13).

The inflammatory environment caused by ovulation involves the release of many components into the surrounding areas, including free radicals, pro-inflammatory cytokines, prostaglandins, and vascular endothelial growth factor (VEGF). The pro-inflammatory cytokine IL-6 plays a role in the pathogenesis of EOC. It has been shown to be associated with worse prognosis, and to promote chemoresistance and stemness (14–16). IL-1 is another pro-inflammatory cytokine released during ovulatory inflammation. The binding of IL-1 to its receptor activates downstream transcription factors activator protein (AP)-1 and nuclear factor kappa B (NF-κB). Both factors play important roles in the inflammatory response and repair during ovulation. They activate many cytokines, chemokines, cell adhesion molecules and, specifically MMP9, which is involved in basement membrane repair (17). However, most recent evidence on the site of origin of HGSOC seems to be leaning away from the OSE.

### The Fallopian Tubes

EOC is known to be developmentally related to the Müllerian duct, an embryological precursor for female reproductive organs, including the fallopian tubes, uterus, cervix and upper vagina, but not the ovaries. An early study, discussing the fallopian tubes as the site of origin of HGSOC rather than the ovary, studied the fallopian tubes from HGSOC patients with BRCA1/2 mutations. It was found that half of these patients presented with dysplasia in the epithelium of the fallopian tubes but not in the ovaries (18). Since then, it has become clear that the fallopian tubes are the most likely sites of origin of HGSOC. Three unique precursor lesions in the fallopian tubes have been identified for HGSOC and will be explored within the following section. These include secretory cell outgrowths (SCOUTs), p53 signature, and serous tubal intraperitoneal carcinoma (STICs) (19) (**Figure 1B**).

The normal fallopian tube epithelium is composed of a mix of secretory and ciliated cells. With age, the number of ciliated cells diminish while secretory cells increase, which has been shown more prominent in patients at high risk for or with HGSOC (20). This is thought to be due to decreases in estrogen levels after menopause, as estrogen stimulates secretion and ciliogenesis in the fallopian tubes (6). These areas of increased secretory cells are termed SCOUTs and are more common in postmenopausal women. SCOUTs are defined as an accumulation of 30 or more secretory cells that can be detected by their low or absent levels of paired box (PAX) 2 (**Figure 1B**). SCOUTs are considered the earliest precursor lesions of HGSOC which PAX2 is reduced in the majority of HGSOCs (21–23). One of the risk factors of HGSOC is elevated estrogen exposure and 80% of HGSOCs have been shown to express estrogen receptor (ER) (24). PAX2 reduction has been associated with increased responsiveness of the ER to estrogen, further supporting the idea that SCOUTs may be precursor lesions of HGSOC (22). Estrogen has been shown to induce tumor formation through the activation of proliferation signaling pathways.

HGSOC can be differentiated from other histotypes of EOC because of its universal TP53 mutations. TP53 signature is a second type of lesion found within the fallopian tube epithelium and thought to be a precursor for HGSOC. It is defined as 10-30 secretory cells with normal appearance that present with an intense p53 immunostaining, suggesting a missense TP53 mutation (**Figure 1B** **)**. These lesions can only be detected by immunostaining as the cellular morphology is identical to that of adjacent normal epithelium (25). A p53 signature has been found predominantly in the secretory cells of the fimbriae, a common site for STIC lesions, and shown to share the same TP53 mutations as STICs. The p53 signature is associated with the cells having double-strand DNA breakage marked by positive γ-H2AX staining (26). Furthermore, inducing p53 mutations in mouse models has been shown to lead to the development of STICs and HGSOC tumors (27). This evidence further supports that the p53 signature may be a precursor lesion for STICs, followed by HGSOC.

STICs are thought to be the immediate precursor lesions of HGSOC. They can be characterized as continuous non-ciliated tubal epithelial cells with nuclear enlargement, loss of cellular polarity, mitotic figures and p53 mutations identical to those found in HGSOC (25, 28). In fact, around 40% of all HGSOCs are accompanied by STIC lesions (29). It is common to observe the presence of both the p53 signature and the STIC lesions in the same patient, suggesting that the p53 signature is likely a precursor to the STICs (30). The majority of STICs have a higher proliferation index than the normal fallopian tube epithelial cells, as noted by elevated level of proliferation marker Ki67. However, 20-30% of STICs were found to have normal levels of Ki67; these cells have been termed dormant STICs and it is unclear whether they remain dormant or can regain proliferative capacity (31, 32).

Continuous ovulation has been shown to be a risk factor for the development of HGSOC. During ovulation, the release of the oocyte is accompanied by follicular fluid, which reaches surrounding tissues, including the fimbriae of the fallopian tubes. This follicular fluid contains reactive oxygen species (ROS) generated because of the damage to the OSE caused by the release of the oocyte. Pro-inflammatory macrophages are also detected at the site of ovulation, which are known to produce ROS (33). The fallopian tube epithelium is exposed to these concentrations of ROS, leading to potential increase in DNA damage. To combat this elevation in ROS, antioxidant systems are activated (34). CD44v9 is a splice variant of the adhesion molecule CD44 and plays a role in redox regulation. For instance, downregulation of CD44v9 is involved in the transformation of clear cell and endometrioid ovarian cancer from endometriosis (35). However, its role in HGSOC is still unclear. One study demonstrated that CD44v9 levels were lower in STICs and HGSOC patient samples compared to normal fallopian tube epithelium, SCOUTs, and the p53 signature, suggesting that a loss of CD44v9 could be involved in the progression from STICs to HGSOC (31). When fallopian tube epithelial cells were exposed to follicular fluid *in vitro*, there was an increase in cell proliferation, induction of double-stranded DNA breaks, and an increase in TP53 mutations (36, 37). Furthermore, the follicular fluid contains insulin-like growth factor (IGF) 2 and IL-8, which have all been shown to increase proliferation (36, 38). IGF-2 was also shown to promote TP53 mutations in fallopian tube epithelial cells (38). Finally, TGFß-1 has been shown to be elevated in STIC lesions and to be produced by tumor-associated macrophages (TAMs). P53 mutant secretory cells were found to induce TGFß1 secretion by TAMs, further supporting the p53 signature as a precursor for STIC **(** **Figure 1B** **)** (39). TGF-ß1 secreted by TAMs has been shown to be involved in EOC progression and will be discussed further throughout this review.

## Microenvironment in the Peritoneal Fluid

A common symptom of late stage metastatic HGSOC is the accumulation of fluid within the peritoneal cavity, which is known as ascites. There are many diseases associated with the abnormal accumulation of peritoneal fluid, including cirrhosis, which accounts for 80% of cases. Cancer is the second leading cause of the presence of ascites fluid and accounts for 10% of cases (40). Malignant ascites accumulation is frequently associated with late-stage ovarian cancer as the volume of ascites fluid increases with disease progression. Furthermore, an increase in ascites fluid has been associated with worse prognosis and increased metastatic potential of ovarian cancer cells (41, 42). The accumulation of ascites fluid associated with ovarian cancer has long thought to be caused by the physical obstruction of lymphatic vessels by tumor cells. However, recent studies have shown that this characteristic accumulation of fluid may also be caused by an increase in vascular permeability due to increased levels of growth factors and cytokines in ascites. This increase in vascular permeability leads to protein leakage, which causes a shift in the osmotic forces leading to an influx of fluid into the peritoneal cavity, contributing to the ascites. The specific components that create a complex microenvironment within this malignant ascites fluid will be discussed in the following section.

### Ascites Fluid

The microenvironment of malignant ascites can be divided into two components: a cellular component and a non-cellular component. The cellular component includes fibroblasts and immune cells, all found floating within the peritoneal fluid, while the acellular component includes cytokines, growth factors, and other metabolites released by the cellular component, surrounding tissues as well as ovarian cancer cells.

#### Cellular Component

**Figure 2:**
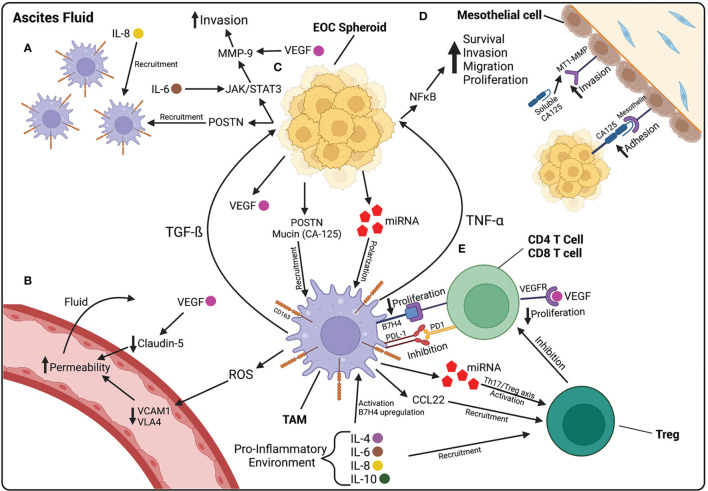
The ascites fluid is composed of cellular and non-cellular components that contribute to the progression of EOC. **(A)** Macrophages are recruited to the peritoneal cavity by IL-8, found at high levels in ascites fluid, and POSTN, released by EOC cells. **(B)** Increased vascular permeability contributes to the accumulation of fluid within the peritoneal cavity. ROS released by macrophages decreases VCAM1 and VLA4, leading to increased permeability. VEGF, released by HGSOC cells, also contributes to increased permeability by decreasing the junctional protein Claudin-5 between endothelial cells. **(C)** EOC spheroids floating in ascites release POSTN and mucins, involved in macrophage recruitment, and miRNA-containing exosomes that polarize macrophages towards a TAM phenotype. TAMs then release TGF-β which activates further POSTN release and the JAK/STAT3 pathway in HGSOC cells, involved in increasing invasion capabilities through MMP-9 activation. TAMs also release TNF-α which activates the NF-κB pathway, involved in increasing the survival, proliferation, migration, and invasion of EOC cells. **(D)** CA125 on the surface of EOC cells plays a role in the adhesion of cancer cells to the mesothelium by binding to mesothelin on the surface of mesothelial cells. CA125 is also found in a soluble form in ascites fluid and increases the expression of MT1-MMP on mesothelial cells, leading to increased invasion of EOC cells. **(E)** TAMs play a role in suppressing the immune system in ascites fluid. Pro-inflammatory cytokines in ascites upregulate B7H4 receptor on TAMs. B7H4 binds to T cells and decreases their proliferation. TAMs also release CCL22, that recruit Tregs, and miRNA containing exosomes, that activate Tregs. Created with BioRender.com.

The ascites fluid that accompanies HGSOC is primarily filled with immune cells and fibroblasts, which play a role in the pathogenesis of ovarian cancer (43). Some of these cells play roles in promoting cancer cell survival and metastasis, while others play a protective role to hinder ovarian cancer progression. In order to survive in ascites fluid, HGSOC cells must avoid immune detection by cells of the innate and adaptive immune system. Both M1 and M2 macrophages are vastly present in ascites fluid. M1 macrophages are pro-inflammatory, have anti-tumor functions, and are classically activated by Th1 cytokines, such as interferon (IFN)-γ. M2 macrophages are activated through an alternate pathway by Th2 cytokines, such as IL-4, IL-10 and IL-13, and function in tissue repair and debris removal; they are also known to have immunosuppressive roles (44). CD163+ tumor-associated macrophages (TAMs) are a subtype of M2 macrophages shown to have tumor promoting functions, including the promotion of tumor growth, survival, and metastasis. They are found in higher frequency in malignant ascites than M1 macrophages and are associated with a higher tumor grade and worse prognosis (45, 46). The pro-inflammatory environment in ascites fluid induces TAMs to their tumor promoting phenotype, specifically through the pro-inflammatory cytokines IL-6 and IL-10 as well as mucins, such as cancer antigen (CA) 125, released by ovarian cancer cells (46, 47). HGSOC cells also release exosomes containing microRNA (miRNA), such as miR-21–3p, miR-125 b-5p and miR-181 d-5p, which induce the polarization of macrophages towards an M2 phenotype (48). HGSOC cells can recruit CD163+ TAMs through the release of periostin (POSTN), which is an ECM protein found to be elevated in ovarian cancer ascites (**Figure 2C**). Once recruited, CD163+ TAMs promote further release of POSTN through transforming growth factor (TGF)-ß, further contributing to the accumulation of TAMs in ascites (49) (**Figure 2A**).

It has been shown that M2 macrophages may be involved in increasing vascular permeability, thereby contributing to the accumulation of ascites fluid. One study demonstrated that M2 macrophages increase vascular permeability through the downregulation of vascular cell adhesion molecule 1 (VCAM1) on endothelial cells and its downstream component very late antigen 4 (VLA4), through an increase in ROS release. Lower VCAM1 and VLA4 in ovarian cancer patient samples was associated with longer survival and lower ascites volumes (50) (**Figure 2B**). Once activated, TAMs release cytokines that activate pro-survival and invasion pathways in HGSOC cells. The increase in NF-κB can induce increased invasiveness of ovarian cancer cells. TNF-α, a cytokine released by TAMs, activates NF-κB pathways (51). TAMs also secrete TGF-ß, leading to the induction of MMPs, further promoting HGSOC invasion. Specifically, TGF-ß released by TAMs induces the activation of the JAK/STAT3 pathway in HGSOC spheroids, leading to the activation of MMP-9, known to be involved in the invasion of HGSOC cells across the ECM (52, 53) (**Figure 2C**).

TAMs play an important immunosuppressive role in the ascites fluid of HGSOC patients. The release of inflammatory cytokines, such as IL-6, IL-10, and TGF-ß, attract regulatory T cells (Treg) and promote their differentiation. Tregs are a subtype of T cells that function as peripheral tolerance checkpoints to suppress activated T cells. They have been shown to be elevated in HGSOC ascites and contribute to T cell tumor-specific immunosuppression (54). As part of their tumor-promoting functions, TAMs recruit Tregs by secreting the chemokine CCL22 (55). TAMs also release exosomes containing miRNA, specifically miR-29a-3p and miR-21-5p, which create an imbalance between Treg and Th17 cells, leaning towards an increase in Tregs (56). IL-6 and IL-10 in the ascites fluid stimulate the expression of B7-H4 co-stimulatory molecule on the surface of TAMs. TAMs that express B7-H4 inhibit T cell proliferation, while blocking B7-H4 expression increases T cell proliferation, demonstrating the presence of a population of immune cell suppressor macrophages (57). In fact, when comparing T cells found in ovarian cancer ascites and T cells in the peripheral blood, T cells found in ascites express higher levels of inhibitory co-receptors, such as LAG-3, PD-1, TIM-3, and CTLA-4, than in the periphery; the expression of these co-receptors is also associated with worse prognosis (58). Both HGSOC cells and TAMs have been shown to express PD-L1, the ligand for PD-1, both contributing to the suppression of T cells and immune escape of HGSOC (59, 60) (**Figure 2E**).

#### Non-Cellular Component

Various acellular factors in ovarian cancer-induced ascites play roles in the regulation of angiogenesis, growth, survival, and metastatic progression of HGSOC cells. These factors include a variety of cytokines, angiogenic factors, and growth factors. Pro-inflammatory cytokines are consistently detected in the ascites fluid of patients with HGSOC (61). Specifically, IL-6, and IL-8, are the most abundant and play important roles in ovarian cancer cell survival, proliferation, adhesion, migration, and invasion (62, 63). IL-6 and IL-8 at high levels in malignant ascites are associated with a worse prognosis and shorter progression-free survival (14, 64). IL-6 is secreted by many cells, including fibroblasts, macrophages, endothelial cells, lymphocytes, and tumor cells, in response to inflammatory signals. This cytokine plays many roles in the activation of adaptive immunity, including activation and differentiation of T lymphocytes and B cells, the recruitment of neutrophils, and the upregulation of adhesion molecules on endothelial cells (65). However, it has also been shown to be involved in the pathogenesis of ovarian cancer. In one study, ascites collected from patients with advanced stage EOC was found to contain high levels of this pro-inflammatory cytokine. IL-6 was also found to increase the activation of Tregs (66) and to enhance the migratory and invasive abilities of ovarian cancer cells through the JAK-STAT3 signaling pathway, involved in cancer progression, and increased EMT (67) (**Figure 2C**).

IL-8 is also a pro-inflammatory chemokine, mainly involved in chemotaxis of immune cells. IL-8 found in patient ascites stimulates angiogenesis. One study showed that ascites rich in cancer cells contained high levels of IL-8, and this ascites was able to stimulate the formation of newer blood vessels (68). The pro-inflammatory environment in ascites fluid contributes to the release of CA-125, also known as mucin 16 (MUC16), by both ovarian cancer cells and mesothelial cells. CA-125 is the most common biomarker for ovarian cancer and is detected at high levels in patients with late-stage chemoresistant disease (69, 70). It is present in both the cell surface and in soluble form. CA-125 binds to mesothelin, a membrane-bound glycoprotein on mesothelial cells, and mediates the binding of ovarian cancer MCSs to mesothelial cells. CA-125, found on the surface of HGSOC cells, binds to mesothelin through the N-glycan domain on CA-125 (71–73). CA-125 also plays a role in the invasion of ovarian cancer cells through the mesothelial monolayer by interacting with MMPs. There seems to be an inverse relationship between the presence of MMPs and CA-125 levels. In fact, one study showed that high levels of membrane type-1(MT1)-MMP, an MMP known to degrade collagen I, correlated with an increase in the soluble form of CA-125 and a decrease in its cell surface form. There was also a reduced adhesion capacity of ovarian cancer cells with the increase of MT1-MMP levels. These results suggest that ovarian cancer cells use surface CA-125 to aid in their adhesion to mesothelial cells, but invasion is potentiated by reduced surface CA-125 and increased soluble CA-125 and MMPs (74) (**Figure 2D**).

VEGF is an angiogenic signaling protein found at elevated levels in ovarian cancer ascites fluid. VEGF ascites levels are higher in patients with a later stage tumor than an early stage and has been associated with a worse prognosis (75, 76). VEGF, released by HGSOC cells, contributes to the accumulation of ascites fluid in ovarian cancer by increasing vascular permeability (77). VEGF was shown to downregulate Claudin 5, a junctional protein involved in endothelial cell intercellular junctions, thus contributing to fluid accumulation (78, 79) (**Figure 2B**). VEGF also increases MMP expression, thereby inducing migratory and invasive behavior in cancer cells (80, 81) (**Figure 2C**). VEGF also reduces the function of immune cells in ascites fluid, thereby contributing to ovarian cancer cell immune evasion. VEGF levels in ascites fluid are inversely proportional to T cell levels (82). One study demonstrated that VEGF is able to suppress proliferation of T cells through the VEGFR-2 receptor (83) (**Figure 2E**). VEGF released by ovarian cancer cells also suppresses the activation of natural killer (NK) cells and their anti-tumor functions (84).

## Ovarian Cancer Spheroids

When ovarian cancer cells detach from their site of origin, either in the ovary or the fallopian tubes, they tend to form MCSs or spheroids within the peritoneal cavity. Spheroids have many advantages over cancer cells travelling individually, including increased survival and ability to adhere and invade at a distant site. The number of spheroids found in ascites increases with advanced disease and has been associated with chemoresistance in patients (85). Spheroids detach from the primary tumor as clusters of cells, rarely mixing amongst each other once in the peritoneal fluid (86). Vitronectin and αv integrins have been observed between ovarian cancer cells in spheroids, and spheroids were unable to form when exposed to blocking antibodies against both adhesion proteins (87). Nectin-4, a cell adhesion molecule, has also been shown to play an important role in spheroid formation. HGSOC cells expressing Nectin-4 form compact spheroids in culture and this effect is inhibited when Nectin-4 is knocked down (88). The composition of ovarian cancer spheroids is still being debated. In many cases, the presence of cancer-associated fibroblasts (CAFs) and TAMs within the spheroids has been reported. One study showed that when ovarian cancer cells were injected into mice, spheroids formed with TAMs located at their center. These TAMs secreted epidermal growth factor (EGF), which increased surface adhesion molecules on both TAMs and ovarian cancer cells, further promoting the adhesion between the two cell types. Elevated EGF levels also increased levels of VEGF and its receptor on ovarian cancer cells, further promoting ovarian cancer cell proliferation and migration (89). A separate study found that spheroids collected from patient samples were PAX8 positive, a marker specific for EOC. In this case, TAMs were mainly located outside the ovarian cancer spheroids. Ovarian cancer cells making up the spheroids were found to have a mixture of epithelial and mesenchymal phenotypes, with mesenchymal cells located more in the center of the clusters (90). There is also evidence that in certain cases, CAFs can be found within the core of HGSOC spheroids. CAFs have been shown to enhance the invasion capacity of HGSOC cells by forming spheroids with cancer cells through integrin α5 and EGF secretion. CAFs lead these spheroids to the peritoneum and help with their survival (91).

Epithelial-to-mesenchymal transition (EMT) is the process leading to a phenotypic change from an epithelial-like cell to a more mesenchymal-like cell. EMT is characterized by the loss of tight junctions between cells, a decrease in E-cadherin (E-Cad), an increase in N-cadherin (N-Cad) expression, and a more migratory and invasive behavior. Ovarian cancer cells seem to undergo EMT when forming spheroids and reverse this process when adhering to a distant site. HGSOC spheroids have reduced E-cad expression compared to the levels found in a primary tumor. This decrease in E-Cad in the spheroids is associated with an increase in mesenchymal markers, such as N-Cad, Snail, Twist1, Twist2, and Zeb2. EMT seems to be initiated by TGF-ß signaling in the peritoneal fluid (92, 93). However, recent evidence has shown that the cadherin profile of ovarian cancer spheroids may not be as straightforward as previously believed. Many studies have demonstrated that spheroids may contain a mix of E-Cad+ and N-Cad+ cells, as well as hybrid cells which are positive for both E-Cad and N-Cad. Differences in the cadherin profile changes the compactness and the aggressiveness of the spheroid, with E-Cad+ dominant spheroids being less compact and aggressive than N-Cad+ dominant spheroids (94, 95). The presence of mixed cadherin and hybrid spheroids suggests differing roles between the various cells within the spheroid. It has been suggested that the N-Cad+ cells provide a metastatic advantage at secondary sites while the E-Cad+ cells provide an advantage during the free-floating stage by promoting resistance to anoikis and chemotherapy (96–99).

Anoikis is a form of apoptotic cell death initiated by the loss of cell adhesion. In order to survive transport by the peritoneal fluid, ovarian cancer spheroids must develop resistance to anoikis. The exact mechanism behind anoikis resistance is still unclear; however, the activation of integrin-dependent pathways seems to be the mechanism behind this resistance to cell death. α5ß3-integrin, a cell adhesion molecule, activates pro-survival pathways and the anti-apoptotic factor Bcl-2. When cells adhere to ECM, α5ß3 integrin activates FAK/PI3K/Akt signaling, which, in turn, promotes the transcription of Bcl-2. Many studies have suggested that this pathway plays an important role in the development of anoikis resistance in ovarian cancer spheroids (100, 101). Protein kinase C (PKC) can also be activated by α5ß3-integrin leading to the activation of the ERK pathway. ERK1/2 phosphorylation has been found to promote ovarian cancer spheroid survival and play a role in the resistance to anoikis (102). A loss of adhesion to ECM can lead to an increase in ROS activation within ovarian cancer cells, which can trigger anoikis. Rho-specific guanine nucleotide exchange factor (Rgnef) is activated downstream of integrin pro-survival pathways and has been shown to reduce ROS activation through NF-κB activation (103).

## Microenvironment at Metastatic Niches

### Cancer-Associated Fibroblasts

**Figure 3:**
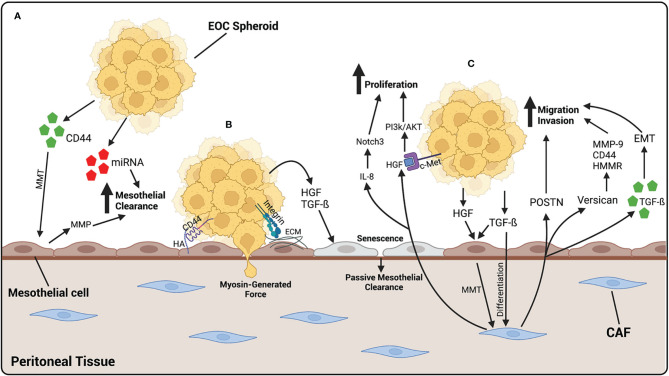
EOC metastasizes to the peritoneal tissue, where it binds and invades through mesothelial cells. **(A)** EOC cells release CD44 which cause MMT of mesothelial cells, giving them a more fibroblast-like function. MMP, released by mesothelial cells, and miRNA-containing exosomes, released by EOC cells, increase mesothelial clearance of cancer cells. **(B)** EOC spheroids adhere to the mesothelium by the binding of CD44 on EOC cells to HA on mesothelial cells or integrins binding to ECM components. Once adhered, EOC cells undergo mesothelial clearance, either by using myosin-generated force, or in a more passive way by generating cellular senescence. HGF and TGF-β released by EOC cells initiates senescence of mesothelial cells, leading to an increase in passive mesothelial clearance. **(C)** Fibroblasts differentiate into CAFs by TGF-β released by EOC cells. HGF and TGF-β released by EOC also contributes to the activation of CAFs, by initiating MMT in mesothelial cells. Once activated, CAFs release POSTN and versican which contribute to the migratory and invasive abilities of EOC cells. CAFs also release exosomes containing TGF-β which increase the ability of cancer cells to undergo EMT, contributing to an increased ability to invade. CAFs also release HGF which binds to c-Met on EOC cells, activating the PI3k/AKT pathway leading to an increase in proliferation. IL-8 released by CAFs activates the Notch3 pathway also leading to an increase in EOC cell proliferation. Created with BioRender.com.

Under normal physiological conditions, fibroblasts play a role in tissue repair and ECM production and secretion. They are activated by tissue damage, becoming highly contractile. These activated fibroblasts are known as myofibroblasts (104). Fibroblasts also play a role in communication between cell types. Recent evidence has shown that fibroblasts communicate with various epithelial cells and immune cells under normal conditions (105). Cancer associated fibroblasts (CAFs) are myofibroblasts that have been activated in association with cancer cells and have been shown to be involved in many cancers, including ovarian cancer (106–109). They play a role in modifying the environment, under the influence of cancer cells, in preparation for metastatic growths and are located in the majority of tissues. CAFs develop the ability to secrete a variety of cytokines and growth factors, reshape the ECM, home cancer cells to a distant site, and aid in their adhesion and migration capabilities (110, 111). CAFs can be recognized by specific biomarkers, including α-SMA, fibroblast-specific protein (FSP1), and fibroblast activation protein α (FAP) (112). The activation of CAFs in HGSOC occurs through the cellular crosstalk between both cancer cells and mesothelial cells. HGSOC cells secrete TGF-ß, which then transform fibroblasts into CAFs (111). Once activated, CAFs promote the migration and invasion of ovarian cancer cells. One study demonstrated that the activation of CAFs by TGF-β leads to the upregulation of versican—an EMC proteoglycan—in fibroblasts, causing an increase in HGSOC motility and invasion (113). This increase in migratory and invasive capabilities was found to be modulated by the activation of hyaluronan-mediated motility receptor (HMMR), MMP-9, and CD44 (113). POSTN, a secretory protein involved in ECM remodeling, has also been shown to increase the migration and invasion of HGSOC cells; it is produced and secreted by CAFs upon activation with TGF-ß (114). In fact, patients with HGSOC associated with high stromal POSTN expression were found to have a worse prognosis, further elucidating the importance of CAFs in ovarian cancer progression (115). CAFs can also originate from mesothelial cells undergoing mesothelial-to-mesenchymal transition (MMT) under the influence of HGSOC cells (116, 117). MMT is the process by which mesothelial cells develop a more fibroblast-like phenotype and acquire the capacity to migrate and invade the submesothelial layer of the peritoneum. This process has been shown to be initiated by the presence of HGSOC and is a way for cancer cells to prepare the environment for easier adhesion and subsequent invasion (118). TGF-ß1 is considered the main factor secreted by HGSOC that initiates the induction of MMT (119, 120). Mesothelial cells exposed to TGF-ß undertake a more mesenchymal phenotype and demonstrate an increase in mesenchymal markers, such as vimentin, α-smooth muscle actin (α-SMA), and N-cadherin (121). The exposure of mesothelial cells to TGF-ß also induces changes that promote peritoneal dissemination and survival of HGSOC cells (122). When exposed to TGF-ß, mesothelial cells increase the production of VEGF, ECM components, and proinflammatory cytokines, while promote the adhesion and proliferation of cancer cells to the peritoneum (120, 121, 123–125). Hepatocyte growth factor (HGF) is another ligand secreted by HGSOC cells involved in the preparation of the pre-metastatic niche through the initiation of MMT (**Figure 3C**). HGF is elevated in HGSOC and high levels are associated with a worse prognosis (126, 127). Furthermore, exposing mesothelial cells to HGF *in vitro* causes them to acquire a spindle-like morphology, lose polarity, and upregulate vimentin expression, as well as an increased ability of cancer cells to invade through them (128).

CAFs have a multitude of functions which promote HGSOC progression, including enhancing tumor growth, ECM remodeling, and cancer cell invasion. HGF is a growth factor released by CAFs that stimulates ovarian cancer cell proliferation, migration, and invasion. The actions of HGF are mediated through the c-Met receptor, found on ovarian cancer cells (129, 130). HGF derived from CAFs promote ovarian cancer cell proliferation through the activation of the PI3K/Akt signaling pathway, favoring cell proliferation and survival, and the upregulation of GRP78, an endoplasmic reticulum chaperone involved in protein folding that plays a role in drug resistance (131) (**Figure 3C**). IL-8 is a cytokine released by CAFs with the ability to promote ovarian cancer cell proliferation and stemness. It does so through the activation of the Notch3 signaling pathway, a pathway known to play many roles in cancer progression (132). CAFs were also shown to release TGF-ß containing exosomes, which can be taken up by ovarian cancer cells and increase their ability to migrate and invade through the initiation of epithelial-to-mesenchymal transition (EMT) (133).

### The Peritoneal Tissue

The peritoneal tissue is a protective layer covering organs within the peritoneal cavity. Once HGSOC spheroids have traveled through and survived the peritoneal fluid, their first point of contact is this tissue. The peritoneal tissue is composed of a sub-mesothelium of fibroblasts embedded in ECM and topped with a monolayer of tightly bound mesothelial cells. Mesothelial cells play a key and complex role in the maintenance of homeostasis within the peritoneal cavity. By producing a coat of glycolipids and glycoproteins on their surface, called the glycocalyx, mesothelial cells provide a non-adhesive and slippery barrier. This barrier acts as both a first line of defense against pathogens and invading tumor cells, as well as facilitates the movement of peritoneal organs.

Mesothelial cells also play a key role in initiating an immune response. Pattern recognition receptors on their surfaces allow mesothelial cells to recognize foreign pathogens, causing them to release inflammatory mediators when activated and initiate an inflammatory response. Inflammatory responses are also initiated after injury to the mesothelial monolayer. Under normal conditions, efficient repair mechanisms initiate a wound healing response that normally takes a few days to resolve. It is when these repair mechanisms are impeded that problems arise, including the adhesion and invasion of metastatic cells (3).

Cell-cell communication between cancer cells and their microenvironment allows for the preparation of the pre-metastatic niche in distant organs and is a crucial part of the establishment of metastatic lesions. The use of exosomes as a means of communication between HGSOC cells and the peritoneal environment has become increasingly relevant and are thought to play a crucial role in facilitating peritoneal metastasis of HGSOC (134). Exosomes are 30–100 nm extracellular vesicles released by a particular cell and can transport a variety of components, including proteins, RNA, and DNA. The contents of exosomes vary greatly depending on the state of the cell releasing them (135). Mesothelial cells are a primary target for exosomes released by HGSOC cells in ascites fluid, as they are the most exposed and therefore the most vulnerable. A common protein released in HGSOC exosomes is CD44, a cell surface adhesion molecule known to bind the receptor hyaluronic acid (HA), which is a key mediator in the adhesion of HGSOC cells to the peritoneum (136–138). CD44 has been shown to be elevated in HGSOC and has been associated with a worse prognosis (139–141). Exosomes containing CD44 function to disrupt the protective mesothelial monolayer and facilitate the adhesion and invasion of HGSOC cells at the peritoneum. One study demonstrated that after an uptake of exosomes released by HGSOC cells, CD44 levels were elevated within mesothelial cells. These mesothelial cells underwent morphological changes and developed a more spindle-like shape. There was also an increase in the secretion of MMPs by mesothelial cells after exposure to CD44-containing exosomes and an increase in mesothelial cell clearance by ovarian cancer cells (142) (**Figure 3A**). In fact, MMP1 mRNA, packaged in exosomes found in patient ascites, is transferred to mesothelial cells and induces apoptosis (143). Another study demonstrates that when exposed to exosomes derived from ovarian cancer malignant-ascites, mesothelial cells were found to have increased abilities to migrate and invade, as well as an increased expression of CAF biomarkers, all characteristics indicative of MMT (144). This change in mesothelial cells towards a more mesenchymal and invasive phenotype has been shown to be possible by the release of various factors through exosomes, including TGF-ß and CD44 (144, 145).

In addition to proteins, miRNAs are also released in exosomes by disseminating cancer cells to prepare metastatic niches. miRNAs are small, non-coding sequences of RNA that have been shown to play roles in gene regulation at the posttranscriptional level and are involved in many diseases, including cancer (146, 147). Specifically, miR-99a-5p was found elevated in exosomes released by HGSOC cells, while treatment of a monolayer of human mesothelial cells with these exosomes increased the ability of HGSOC cells to invade through by increasing the expression of ECM components (148). MiR-21 and miR-29a, also found in exosomes released by HGSOC cells, increase mesothelial clearance *in vitro* as well as correlate with lower survival in patients (149) (**Figure 3A**).

The adhesion, migration, and invasion of HGSOC cells to the peritoneum is carried out by changes in cellular fibrillar actin structure and the formation of filopodia, lamellipodia, and invadopodia. In order to adhere, cancer cells modulate the number and type of adhesion molecules on their surfaces. Many adhesion molecules are involved in the adhesion of HGSOC cells to the mesothelial monolayer. As previously discussed, CD44 is an adhesion molecule found on ovarian cancer cells having high affinity for hyaluronic acid; the interaction between the two is thought to be a key modulator of HGSOC cell adhesion (**Figure 3B**). However, there are multiple adhesion molecules involved in the adhesion of HGSOC cells to the mesothelial monolayer. Selectins are glycoproteins involved in cell-cell adhesion under hydrodynamic flow, most known for their role in the adhesion of leukocytes to endothelial cells when traveling to a site of injury or infection (150). It is now clear that selectins may also be involved in the adhesion of HGSOC cells to mesothelial cells, since the dissemination of cancer cells in the peritoneal fluid is a dynamic process, similar to that of leukocytes traveling in the blood stream. A study demonstrated that E-, P- and L-selectins were all expressed on human peritoneal mesothelial cells and treatment with blocking antibodies for each of the adhesion molecules slowed the rate of adhesion of metastatic ovarian cancer cells under flow. Interestingly, inhibition of P-selectin almost completely inhibited the adhesion of ovarian cancer cells to the mesothelial cell monolayer, compared to inhibition of S- and L-selectins, which partially slowed the adhesion rate (151). Further emphasizing the role of P-selectin in HGSOC cell adhesion, another study demonstrated that macrophages exposed to HGSOC cells increased the adhesion of cancer cells to mesothelial cells by upregulating P-selectin molecules on the mesothelial cell surface (152).

Other adhesion molecules thought to play a role in HGSOC cell-mesothelial cell adhesion are CA125 and CD133. CA125 is primarily known for its role as a prognosis marker in ovarian cancer patients. However, CA125 can also be found on the surface of ovarian cancer cells and acts as an adhesion molecule, binding to mesothelin on the mesothelial cell surface (72–74) (**Figure 2D**).

Integrins are a class of cell adhesion molecules with high affinity for ECM components. They play a role in the adhesion of EOC spheroids to the mesothelial monolayer. Specifically, α5ß1 integrin is involved in the binding of EOC spheroids to fibronectin, found in the peritoneal stroma (153). In fact, α5ß1 integrins are commonly expressed in malignant EOC cells collected from patient pleural effusions, further elucidating their role in EOC metastasis (154). Moreover, α2ß1 integrin plays a role in the disaggregation of EOC spheroids followed by an increased adhesion to collagen I, an ECM protein EOC cells preferentially bind to (155, 156) **(** **Figure 2D**).

Mesothelial clearance is the process in which ovarian cancer cells displace mesothelial cells in order to invade into the underlying stroma to form metastatic lesions. The specific mechanism of invasion through the mesothelium is still under question. It was long thought that HGSOC cells infiltrate through the mesothelial monolayer by generating force, mediated by myosin, in order to migrate through the intercellular spaces between mesothelial cells (157) (**Figure 3B**). Filopodia have been shown to be key players in trans-mesothelial migration of HGSOC cells, elucidating the importance of cancer cell adhesion to the mesothelial cell monolayer in order for invasion to occur. In one study, downregulation of the filopodia proteins fascin-1 and myosin-X lead to an inhibition of trans-mesothelial migration *in vitro* and the adherence of cancer cells to mesothelial cells *in vivo* (158).

More recent studies, however, have demonstrated that invasion through the mesothelium may be a more passive process, during which activated mesothelial cells migrate away from HGSOC cells, providing space for them to adhere and invade (159). One study demonstrated that exposure to malignant ascites facilitated ovarian cancer trans-mesothelial migration and reduced the expression of junctional proteins between mesothelial cells (160). Recent evidence has shown that senescent mesothelial cells may encourage the trans-mesothelial migration of HGSOC cells in a more passive manner (**Figure 3B**). This theory was first suggested after the observation of invaded cancer cells found below an intact mesothelial monolayer (161, 162). Cellular senescence occurs with age and is characterized by a cell’s inability to divide. Cellular senescence is often associated with cell-cycle arrest in the G1 phase and increased expression of senescence-associated ß-galactosidase (SA-ß-Gal). However, senescent cells remain alive and continue to have the ability to secrete many molecules which influence their environment (163). Ovarian cancer cells exposed to senescent mesothelial cells have a higher proliferation rate, adhesion to mesothelial cells, and invasive capacity across a monolayer of mesothelial cells *in vitro*, as well as an increased ability to develop tumors *in vivo* (164, 165). An increase in senescent mesothelial cells has been associated with age and could explain the increase in aggressivity of metastatic lesions found in older patients with HGSOC (166).

The passive invasion of HGSOC cells through the mesothelial cell monolayer involves the downregulation of intracellular junction proteins between mesothelial cells. Four junctional proteins, connexin 43, E-cadherin, occludin, and desmoglein, were found to be decreased in senescent mesothelial cells compared to their younger counterparts. When exposed to neutralizing antibodies for TGF-ß and HGF, the loss of each of the junctional proteins was inhibited in senescent mesothelial cells as well as the trans-mesothelial invasion of ovarian cancer cells (161). Regardless of the exact process, many biological markers have been identified as promoting mesothelial clearance by HGSOC cells, including keratin-14, ADH1B, and mesothelin (167–169).

### The Omentum

**Figure 4:**
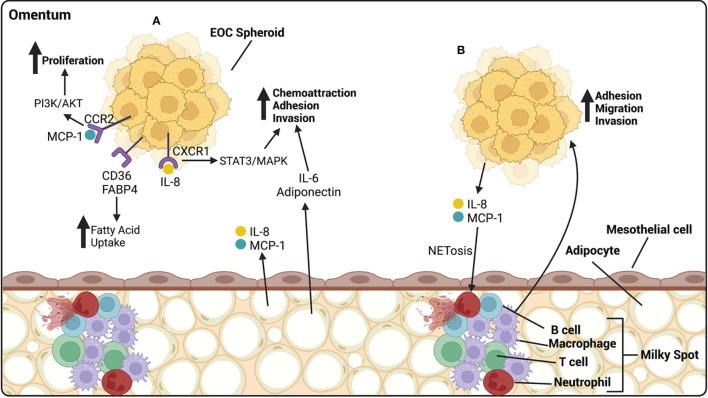
EOC metastasize preferentially to the omentum, composed of mesothelial cells atop of adipocytes. **(A)** Adipocytes release cytokines and chemokines that influence the progression of EOC. IL-6 and adiponectin act as chemoattractants, homing HGSOC spheroids to the omentum, and increasing their ability to adhere and invade. IL-8, released by adipocytes, binds CXCR1 on EOC cells, activating the STAT3/MAPK pathway and increasing adhesion and invasion of EOC cells. MCP-1, released by adipocytes, binds CCR2 on EOC cells, activating the PI3k/AKT pathway, increasing proliferation. CD36 and FABP4 receptors on EOC cells are involved in the uptake of fatty acids that can be utilized by the cancer cells as a source of energy. **(B)** Milky spots are highly vascularized areas, rich in immune cells. They are primarily composed of macrophages with a lesser proportion of T cells, B cells and neutrophils. EOC cells release IL-8 and MCP-1 that cause NETosis of neutrophils, to which cancer cells preferentially bind. Macrophages release many cytokines aiding in the adhesion, migration and invasion of HGSOC spheroids. Created with BioRender.com.

The omentum is a large fatty tissue that covers the stomach and the bowel. It is a common site of metastasis for HGSOC and 80% of all patients will present with metastatic lesions in the omentum. The omentum is composed of mesothelial cell layers enclosing adipocytes and connective tissue. It is filled with milky spots, which are areas comprised of a rich capillary network and an accumulation of immune cells, the majority of them being macrophages, with a smaller proportion of B and T cells. The omentum functions as a lipid storage site, as well as an endocrine organ. It also has a defensive role and is the main source of bacteria and debris clearance from the peritoneal cavity (170). Adipocytes increase the ability of ovarian cancer cells to migrate and invade. Adipocytes secrete a vast array of cytokines including IL-6, IL-8, monocyte chemoattractant protein-1 (MCP-1), and adiponectin, which home ovarian cancer cells to the omentum and encourage their adhesion and invasion to the organ. IL-8 binds CXCR1 on ovarian cancer cells and promotes metastasis through activation of STAT3 and p38 MAPK pathways (171). MCP-1 binds to CCR-2 on the surface of ovarian cancer cells and initiates migration through the PI3K/AKT pathway (172). CXCR2 is a chemokine secreted by adipocytes and has been associated with increased tumor burden *in vivo* compared to knock out animals, which had higher levels of ovarian cancer cells in ascites. CXCR2 is associated with obesity, a known risk factor for HGSOC (173) (**Figure 4A**).

Exposure of ovarian cancer cells to adipocytes leads to lipid accumulation within the cancer cells. These lipids are utilized by the cancer cells as a source of energy through ß-oxidation, which increases the proliferation capacity of the cells (171, 174). CD36 is a fatty acid receptor found to be elevated in ovarian cancer cells cultured with adipocytes, allows for cellular uptake of fatty acids, and causes a metabolic reliance on exogenous lipids by reducing the glucose oxidation capabilities of tumor cells (175). Exposure to the inflammatory cytokine IL-17 has been shown to upregulate FABP4 on ovarian cancer cells, leading to an increased uptake of fatty acids. IL-17 also activates the STAT3 pathway, inducing proliferation of ovarian cancer cells (176) (**Figures 4A**).

Omental milky spots are major defenders within the peritoneal cavity. Ovarian cancer cells preferentially metastasize to omental fatty tissue that contains milky spots rather than tissue without (177, 178). While neutrophils make up only about 1% of cells within the peritoneal fluid, their influx into the milky spots is a critical step for the initiation of ovarian cancer metastasis to the omentum. Once at the omentum, neutrophils undergo a specialized form of cell death called NETosis during which they extrude chromatin fibers called neutrophil extracellular traps (NETs). NETosis is initiated by the release of inflammatory cytokines by ovarian cancer cells, including IL-8 and MCP-1. Ovarian cancer cells will preferentially bind to NETs in the omentum (179). Also CD163+ TAMs have been found within milky spots and have been shown to promote metastasis of ovarian cancer cells to the omentum through increases in EMT and cancer stem cell markers (180). They also promote the migration of ovarian cancer cells to the omentum through chemokine release that interact with chemokine receptor 1 (CCR1) on ovarian cancer cells and associate with activation of ERK1/2 and PI3K pathways (181) (**Figure 4B**).

Crown-like structures (CSCs) are characterized as dying adipocytes surrounded by rings of macrophages and are a hallmark of an inflammatory environment. The presence of CSCs and an accumulation of CD163+ TAMs in the omentum in patients with advanced HGSOC has been associated with a worse prognosis. Interestingly, patients with advanced stage HGSOC but low levels of CD163+ TAMs show no association with patient prognosis, implying a critical role of TAMs in HGSOC progression and aggressivity within the omentum (182).

## Microenvironment and Chemoresistance

The current treatment for HGSOC involves debulking surgery to remove any macroscopic masses, followed by platinum-based combination chemotherapy. The platinum-based agents approved in the clinic, cisplatin, carboplatin, and oxaliplatin, induce toxicity in ovarian cancer cells by generating DNA crosslinking, leading to DNA damage and triggering apoptosis. This treatment has remained unchanged since the approval of cisplatin in the 1980s followed by carboplatin in 1989, and oxaliplatin in 2000, even though the development of chemoresistance disease occurs in approximately 95% of patients. The 5-year survival rate after treatment has remained at less than 40% for the past three decades, creating a desperate need for new therapeutic options for this disease (1). There are three mechanisms known to promote chemoresistance in HGSOC: the reduction of intracellular influx of platinum, the control of DNA repair pathways, and the blocking of DNA damage-induced cell death (183). The complex microenvironment plays a large role in the progression of HGSOC as well as the development of chemoresistance through each one of these mechanisms.

The microenvironment in ascites fluid contains immune cells, reprogrammed to have tumor-promoting functions. HGSOC cells treated with cisplatin have been shown to increase the polarization of macrophages towards a TAM phenotype by releasing IL-6 and PGE2 inflammatory cytokines. These TAMs showed increased expression of STAT3, known to promote tumor survival, and a decrease in STAT6 and STAT1, which are immune potentiators (184). A microenvironment composed of TAMs can further contribute to a chemoresistant phenotype in ovarian cancer. TAMs have been shown to release exosomes containing miR-223 that promotes drug resistance in ovarian cancer cells by blocking PTEN expression, a protein known to block cell survival pathways. This reduction allows for the increased expression of the PI3K/AKT cell survival pathway (185).

CAFs play important roles in tumor progression as well as immune regulation and a multitude of evidence points to a potential role in the development of chemoresistance in HGSOC. CAFs reduce the uptake of cisplatin by HGSOC cells by increasing intracellular glutathione (GSH) levels. CAFs release high levels of GSH, which is then taken up by HGSOC cells. GSH can bind internalized cisplatin and the GSH-cisplatin complex effluxes from the cell. CD8+ T cells were found to counteract this effect by promoting GSH degradation through IFN-γ release (186). CAFs were also found to induce EMT, through the activation of the JAK/STAT3 pathway, in ovarian cancer cells by secreting IL-6 the JAK/STAT3 pathway leads to a decrease in platinum-induced apoptosis due to a decrease in the pro-apoptotic protein Bax and caspase-3 (187, 188). Interestingly, ECM stiffness also plays a role in platinum resistance. HGSOC cells were found to be more resistant to chemotherapy when adhered to stiff substrate compared to a soft substrate. Specifically, substrates composed of collagen-6 and fibronectin were found to induce chemoresistance caused by an increase in integrin activation (189).

HGSOC cells influence the pro-tumor functions of mesothelial cells through the release of TGF-ß. Osteopontin (OPN) is a glycoprotein released by mesothelial cells under the influence of TGF-ß and potentiates chemoresistance in ovarian cancer cells. It signals through the binding of CD44 and integrins on cancer cells and activates PI3k/AKT tumor survival pathway and the expression of ATP-binding cassette (ABC) transporters involved in drug efflux, a similar mechanism to the upregulation of copper transporters ATP7A and ATP7B that function to efflux platinum from the cell leading to chemoresistance (190, 191). Another study showed that the upregulation of fibronectin on the surface of mesothelial cells, induced by TGF-ß, caused chemoresistance in ovarian cancer cells *via* the PI3K/AKT pathway (119). This study suggests a similar mechanism in which the binding of cell surface adhesion molecules on ovarian cancer cells activates a pro-survival signaling pathway.

Adipocytes secrete arachidonic acid (AA), a fatty acid that induces chemoresistance in HGSOC cells by activating the PI3K/AKT survival pathway, thereby blocking apoptosis (192). Adipocytes release exosomes containing miRNAs, which can induce platinum resistance in ovarian cancer cells. miR21, found in exosomes released by both adipocytes and CAFs, downregulate APAF1, a protein found to be involved in chemoresistance and reduced apoptosis (193)

Finally, HGSOC cells become chemoresistant when placed under the fluidic stress within the ascitic fluid. Ovarian cancer cells treated with cisplatin and paclitaxel under a microfluidic model had a higher viability than those treated under static conditions. Chemoresistance induced by fluid stress has been associated with activation of PI3K/Akt pathway and EGFR (194, 195).

## Conclusion

The transcoelomic ecosystem in EOC plays a crucial role in the progression of the disease. The pro-inflammatory environment in both the OSE and the fallopian tubes provides opportunities for the accumulation of DNA damage, potentially leading to the formation of precursor lesions with increased proliferative capacities. The lack of anatomical barriers between the ovaries, fallopian tubes, and the fluid-filled peritoneal cavity allows for primary EOC tumors to influence the microenvironment within the peritoneal fluid and facilitate their dissemination and survival. EOC cells form spheroids that provide them with the ability to avoid immune detection and to resist to anoikis, while secreting factors that activate the tumor-supporting functions of surrounding macrophages. Cell-cell communication between EOC cells and cells within the peritoneal ecosystem further promotes the survival and dissemination of EOC, in particular to the peritoneal tissue and the omentum. Fibroblasts, mesothelial cells and adipocytes play crucial roles in inducing adhesion and invasion at distant metastatic sites. Finally, the cellular players involved in EOC dissemination all play a further role in the development of chemoresistance, a common occurrence in ovarian cancer. The understanding of the transcoelomic ecosystem in EOC is important in creating new therapeutic targets and furthering the development of novel treatment options for this devastating disease.

## Author Contributions

SR drafted the manuscript and compiled the figures. CT supervised the graduate student and revised and approved the final version of the manuscript.

## Funding

This research was funded through the OvCAN initiative of Ovarian Cancer Canada.

## Conflict of Interest

The authors declare that the research was conducted in the absence of any commercial or financial relationships that could be construed as a potential conflict of interest.

## Publisher’s Note

All claims expressed in this article are solely those of the authors and do not necessarily represent those of their affiliated organizations, or those of the publisher, the editors and the reviewers. Any product that may be evaluated in this article, or claim that may be made by its manufacturer, is not guaranteed or endorsed by the publisher.

## Abbreviations

AA, Arachidonic acid; CA, Cancer antigen; CAF, Cancer associated fibroblast; CCR1, Chemokine receptor 1; CSC, Crown-like structures; E-Cad, E-cadherin; ECM, Extracellular matrix; EMT, Epithelial-to-mesenchymal transition; EOC, Epithelial ovarian cancer; ER, Estrogen receptor; GSH, Glutathione; HA, Hyaluronic acid; HGF, Hepatocyte growth factor; HGSOC, High grade serous ovarian cancer; IGF, Insulin-like growth factor; IFN, Interferon.

IL, Interleukin; MCP, Monocyte chemoattractant protein; MCS, Multicellular structures; miRNA, MicroRNA; MMP, Matrix metalloproteinase; MMT, Mesothelial-to-mesenchymal transition; MUC16, Mucin 16; N-Cad, N-cadherin; NET, Neutrophil extracellular traps; NF-κB - Nuclear factor kappa B; OPN, Osteopontin; OSE, Ovarian surface epithelium; PAX, Paired box; POSTN, Periostin; Rgnef - Rho-specific guanine nucleotide exchange factor; ROS, Reactive oxygen species; SCOUT, Secretory cell outgrowth; SMA, Smooth muscle actin; STIC, Serous tubal intraperitoneal carcinoma; TAM, Tumor-associated macrophage; TGF, Transforming growth factor; TNF, Tumor necrosis factor; Treg, Regulatory T cell; uPA, Urokinase type plasminogen activator; VCAM, Vascular cell adhesion molecule; VEGF, Vascular endothelial growth factor; VLA, Very late antigen.
